# Association of abnormal explicit sense of agency with cerebellar impairment in myoclonus-dystonia

**DOI:** 10.1093/braincomms/fcae105

**Published:** 2024-03-27

**Authors:** Clément Tarrano, Cécile Galléa, Cécile Delorme, Eavan M McGovern, Cyril Atkinson-Clement, Isaac Jarratt Barnham, Vanessa Brochard, Stéphane Thobois, Christine Tranchant, David Grabli, Bertrand Degos, Jean Christophe Corvol, Jean-Michel Pedespan, Pierre Krystkowiak, Jean-Luc Houeto, Adrian Degardin, Luc Defebvre, Romain Valabrègue, Benoit Beranger, Emmanuelle Apartis, Marie Vidailhet, Emmanuel Roze, Yulia Worbe

**Affiliations:** CNRS UMR 7225, Sorbonne Université, Paris Brain Institute—Institut du Cerveau et de la Moelle épinière, Inserm U1127, Paris 75013, France; Assistance Publique-Hôpitaux de Paris, Pitié-Salpêtrière Hospital, Department of Neurology, Clinical Investigation Center for Neurosciences, Paris 75013, France; CNRS UMR 7225, Sorbonne Université, Paris Brain Institute—Institut du Cerveau et de la Moelle épinière, Inserm U1127, Paris 75013, France; Department of Research Neuroimaging, Centre de NeuroImagerie de Recherche (CENIR), Sorbonne Université, Paris 75013, France; CNRS UMR 7225, Sorbonne Université, Paris Brain Institute—Institut du Cerveau et de la Moelle épinière, Inserm U1127, Paris 75013, France; Assistance Publique-Hôpitaux de Paris, Pitié-Salpêtrière Hospital, Department of Neurology, Clinical Investigation Center for Neurosciences, Paris 75013, France; Department of Neurology, Beaumont Hospital, Dublin 9, D09 VY21, Ireland; School of Medicine, Royal College of Surgeons in Ireland, Dublin 2, D02 YN77, Ireland; CNRS UMR 7225, Sorbonne Université, Paris Brain Institute—Institut du Cerveau et de la Moelle épinière, Inserm U1127, Paris 75013, France; School of Medicine, University of Nottingham, Nottingham NG7 2RD, UK; Department of Psychiatry, University of Cambridge, Cambridge CB2 0SZ, UK; Assistance Publique-Hôpitaux de Paris, Pitié-Salpêtrière Hospital, Department of Neurology, Clinical Investigation Center for Neurosciences, Paris 75013, France; Department of Neurology, Hospices Civils de Lyon, Lyon 69000, France; Département de Neurologie, Hôpitaux Universitaires de Strasbourg, Hôpital de Hautepierre, Strasbourg 67098, France; INSERM-U964/CNRS-UMR7104, Institut de Génétique et de Biologie Moléculaire et Cellulaire (IGBMC), Université de Strasbourg, Illkirch 67404, France; Fédération de Médecine Translationnelle de Strasbourg (FMTS), Université de Strasbourg, Strasbourg 67000, France; CNRS UMR 7225, Sorbonne Université, Paris Brain Institute—Institut du Cerveau et de la Moelle épinière, Inserm U1127, Paris 75013, France; Assistance Publique-Hôpitaux de Paris, Pitié-Salpêtrière Hospital, Department of Neurology, Clinical Investigation Center for Neurosciences, Paris 75013, France; Department of Neurology, Assistance Publique-Hôpitaux de Paris, Avicenne Hospital, Sorbonne Paris Nord, Bobigny 93000, France; CNRS UMR 7225, Sorbonne Université, Paris Brain Institute—Institut du Cerveau et de la Moelle épinière, Inserm U1127, Paris 75013, France; Assistance Publique-Hôpitaux de Paris, Pitié-Salpêtrière Hospital, Department of Neurology, Clinical Investigation Center for Neurosciences, Paris 75013, France; Department of Neuropediatry, Universitary Hospital of Pellegrin, Bordeaux 33076, France; Department of Neurology, Abu Dhabi Stem Cells Centre, Abu Dhabi, United Arab Emirates; Department of Neurology CHU Limoges, Inserm U1094, IRD U270, Univ. Limoges, EpiMaCT—Epidemiology of chronic diseases in tropical zone, Institute of Epidemiology and Tropical Neurology, OmegaHealth, Limoges 87000, France; Department of Neurology, Tourcoing Hospital, Tourcoing 59599, France; Department of Neurology, University of Lille, Lille 59000, France; Department of Neurology, Lille Centre of Excellence for Neurodegenerative Diseases » (LiCEND), Lille F-59000, France; CNRS UMR 7225, Sorbonne Université, Paris Brain Institute—Institut du Cerveau et de la Moelle épinière, Inserm U1127, Paris 75013, France; Department of Research Neuroimaging, Centre de NeuroImagerie de Recherche (CENIR), Sorbonne Université, Paris 75013, France; CNRS UMR 7225, Sorbonne Université, Paris Brain Institute—Institut du Cerveau et de la Moelle épinière, Inserm U1127, Paris 75013, France; Department of Research Neuroimaging, Centre de NeuroImagerie de Recherche (CENIR), Sorbonne Université, Paris 75013, France; CNRS UMR 7225, Sorbonne Université, Paris Brain Institute—Institut du Cerveau et de la Moelle épinière, Inserm U1127, Paris 75013, France; Department of Neurophysiology, Saint-Antoine Hospital, Paris 75012, France; CNRS UMR 7225, Sorbonne Université, Paris Brain Institute—Institut du Cerveau et de la Moelle épinière, Inserm U1127, Paris 75013, France; Assistance Publique-Hôpitaux de Paris, Pitié-Salpêtrière Hospital, Department of Neurology, Clinical Investigation Center for Neurosciences, Paris 75013, France; CNRS UMR 7225, Sorbonne Université, Paris Brain Institute—Institut du Cerveau et de la Moelle épinière, Inserm U1127, Paris 75013, France; Assistance Publique-Hôpitaux de Paris, Pitié-Salpêtrière Hospital, Department of Neurology, Clinical Investigation Center for Neurosciences, Paris 75013, France; CNRS UMR 7225, Sorbonne Université, Paris Brain Institute—Institut du Cerveau et de la Moelle épinière, Inserm U1127, Paris 75013, France; Department of Neurophysiology, Saint-Antoine Hospital, Paris 75012, France

**Keywords:** sense of agency, myoclonus dystonia, MRI, cerebellum, metacognition

## Abstract

Non-motor aspects in dystonia are now well recognized. The sense of agency, which refers to the experience of controlling one's own actions, has been scarcely studied in dystonia, even though its disturbances can contribute to movement disorders. Among various brain structures, the cerebral cortex, the cerebellum, and the basal ganglia are involved in shaping the sense of agency. In myoclonus dystonia, resulting from a dysfunction of the motor network, an altered sense of agency may contribute to the clinical phenotype of the condition. In this study, we compared the explicit and implicit sense of agency in patients with myoclonus dystonia caused by a pathogenic variant of *SGCE* (DYT-*SGCE*) and control participants. We utilized behavioural tasks to assess the sense of agency and performed neuroimaging analyses, including structural, resting-state functional connectivity, and dynamic causal modelling, to explore the relevant brain regions involved in the sense of agency. Additionally, we examined the relationship between behavioural performance, symptom severity, and neuroimaging findings. We compared 19 patients with DYT-*SGCE* and 24 healthy volunteers. Our findings revealed that patients with myoclonus-dystonia exhibited a specific impairment in explicit sense of agency, particularly when implicit motor learning was involved. However, their implicit sense of agency remained intact. These patients also displayed grey-matter abnormalities in the motor cerebellum, as well as increased functional connectivity between the cerebellum and pre-supplementary motor area. Dynamic causal modelling analysis further identified reduced inhibitory effects of the cerebellum on the pre-supplementary motor area, decreased excitatory effects of the pre-supplementary motor area on the cerebellum, and increased self-inhibition within the pre-supplementary motor area. Importantly, both cerebellar grey-matter alterations and functional connectivity abnormalities between the cerebellum and pre-supplementary motor area were found to correlate with explicit sense of agency impairment. Increased self-inhibition within the pre-supplementary motor area was associated with less severe myoclonus symptoms. These findings highlight the disruption of higher-level cognitive processes in patients with myoclonus-dystonia, further expanding the spectrum of neurological and psychiatric dysfunction already identified in this disorder.

## Teaser

Tarrano *et al*. report that in myoclonus dystonia, impaired explicit agency correlates with structural abnormalities in the motor cerebellum and altered connectivity with the pre-supplementary motor area. Pre-supplementary motor area activity also correlates with myoclonus severity, emphasizing the cerebellum's role in both motor and non-motor expressions of myoclonus dystonia.

## Graphical Abstract

**Graphical abstract:**
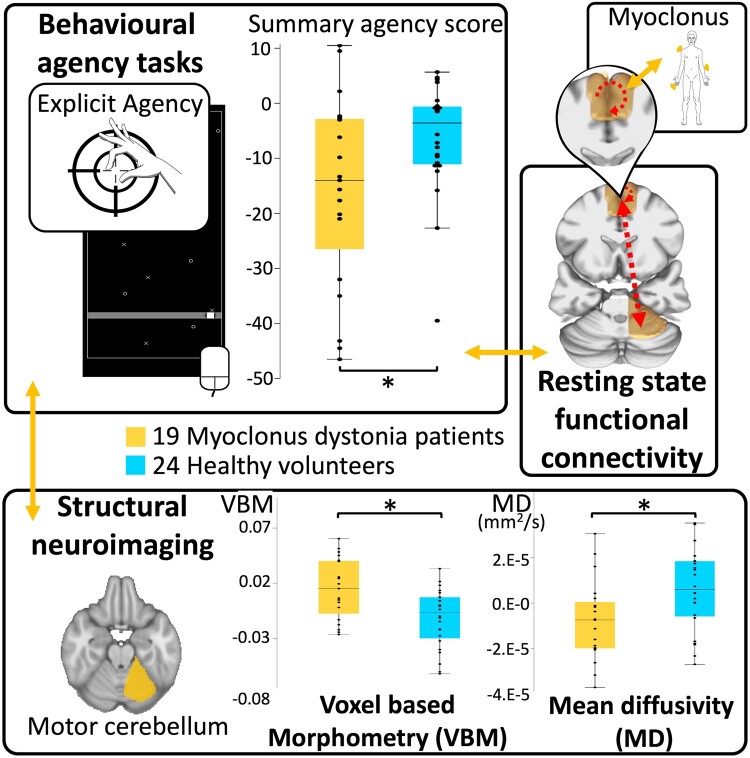

## Introduction

Sense of agency (SoA) is a cognitive function related to actions and defined as the experience of controlling one's own actions and the events they produce in the external world.^1^ SoA plays a crucial role in adjusting movements and behaviours in response to environmental stimuli and optimizing motor control.^2^ Movement disorders can lead to a loss of control during voluntary actions, and this loss of control has been associated with disruptions in SoA in conditions such as Parkinson's disease, Tourette's disorder and functional neurological disorders.^3-8^ However, the exact relationship between alterations in SoA, movement disorders and dysfunction in brain networks remains unclear.^3^ For example, the motor network has been implicated in both explicit SoA (the subjective feeling of action causality) and implicit SoA (objective psychometric measures reflecting SoA).^1,9^ Studies of explicit SoA, using paradigms involving sensorimotor incongruency, have linked the pre-supplementary motor area (pre-SMA) and supplementary motor area (SMA proper) to sensorimotor mismatch detection.^9,10^ The putamen and motor cerebellum have been identified as contributing to SoA by evaluating action-outcome predictions.^9^ Specifically, during self-generated actions, the cerebellum and its forward model are involved in predicting feedbacks,^9^ while the basal ganglia are involved in predicting rewards.^11^ Predictions constitute a fundamental component in the construction of the sense of agency.^1^ In addition to the motor system, the temporo-parietal junction [particularly the inferior parietal lobule (IPL)] has been associated with detecting action-outcome mismatches, while the insula and precuneus have been linked to detecting sensorimotor congruency.^9^ Studies investigating implicit SoA have shown that the cingulate motor area and the pre-SMA are associated with attentiveness to action onset and intention.^9,12,13^

Dystonia is a movement disorder that involves several brain regions implicated in the generation of SoA, including the pre-SMA, IPL, cerebellum and basal ganglia.^14-16^ Previous research has identified abnormal SoA in patients with idiopathic adult-onset cervical dystonia.^17^ In this study, we aim to explore the relationship between SoA and underlying dysfunction in brain networks in another form of dystonia known as myoclonus-dystonia. Myoclonus-dystonia is a neurodevelopmental disorder associated with variations in the sarcoglycan epsilon (*SGCE*) gene (DYT-*SGCE*) and is characterized by childhood-onset dystonia and sub-cortical myoclonus predominantly affecting the upper body.^18,19^ DYT-*SGCE* is also more frequently associated with psychiatric comorbidities compared to other forms of hereditary dystonia.^20,21^ The clinical manifestations of DYT-*SGCE* arise from a dysfunction of the motor network, encompassing the cerebellum, basal ganglia and the motor cortex.^22-30^ DYT-*SGCE* dystonia differs significantly from idiopathic adult-onset cervical dystonia in terms of clinical phenotype and onset. It remains unclear whether abnormal SoA represents a common feature across various forms of dystonia. It remains unclear whether abnormal SoA represents a common feature across various forms of dystonia.

To further identify the spectrum of cognitive dysfunction in DYT-*SGCE*, we hypothesize that patients exhibit altered behavioural measures related to the SoA, which are associated with structural and functional network deficits. To test this hypothesis, we assessed SoA alterations using both explicit and implicit measures of agency in behavioural tasks.^31^ These tasks and various conditions were designed to separately test the multiple components involved in constructing the sense of agency. The advantage of such an approach was to discuss brain networks dysfunction in DYT-*SGCE* in relation to the distinct components of SoA alterations in various tasks and conditions. If the cerebellum and basal ganglia, which play a key role in predicting action outcomes and motor adaptation, were predominantly affected, the sense of agency in these situations would be selectively altered in DYT-*SGCE*. Conversely, if the alterations were more widespread, encompassing multimodal cortical regions, the sense of agency would likely be altered on multiple levels in these patients and independent of action-outcome adaptations. To assess the impact of these changes, we conducted structural and resting-state functional MRI studies to investigate the morphology and functional connectivity among relevant brain regions involved in SoA construction. Furthermore, we examined the relationships between task performance and neuroimaging findings.

## Materials and methods

### Subjects

DYT-*SGCE* subjects were recruited through the French Movement Disorders Clinics Network and clinically evaluated at the Pitié-Salpêtrière Hospital in Paris. Healthy volunteers were recruited through advertisement via a local neuroscience research platform. Subjects’ consent was obtained according to the Declaration of Helsinki, and Ethics committee approval was obtained (CPP/AU-1360).

Patients’ inclusion criteria were: (i) aged between 15 and 60 years old (ii) clinical diagnosis of myoclonus-dystonia with *SGCE* mutation, (iii) absence botulinum toxin injection for at least three months prior to study participation, (iv) absence of pharmacological treatment modification for at least 1 month prior to the study, (v) absence of MRI contraindication including deep brain stimulation. Healthy subjects were matched to DYT-*SGCE* for age, sex, educational level, handedness and had no history of chronic illness or habitual use of medication (except for oral contraception for females).

Disease severity was assessed using the Burke-Fahn-Marsden scale (BFM) for dystonia and the Unified Myoclonus Rating Scale (UMRS) for myoclonus.^32,33^ All subjects were screened for psychiatric comorbidities using the Mini International Neuropsychiatric Interview (MINI).^34^

### Behavioural tasks

#### Explicit agency task

**Figure 1:**
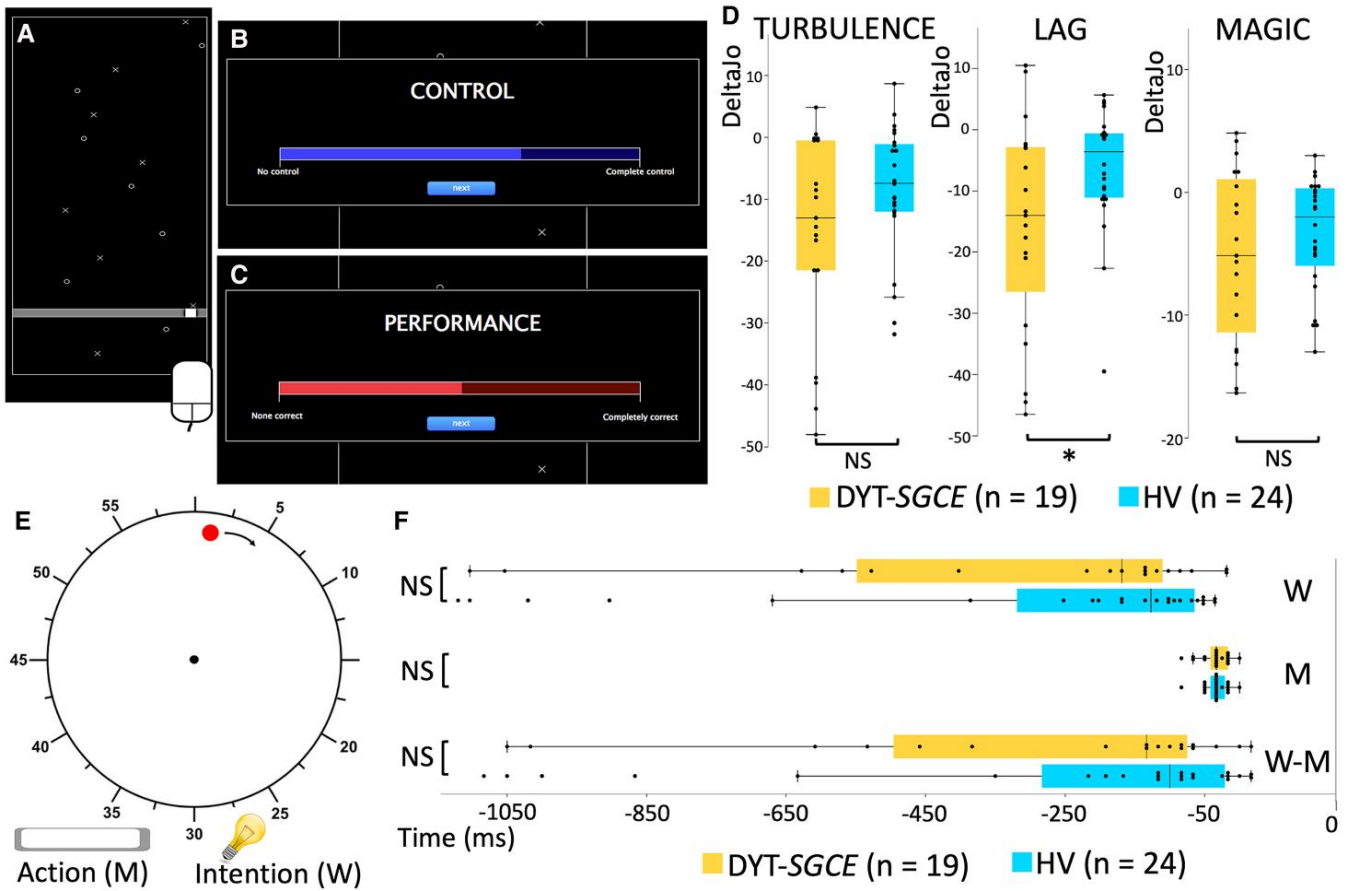
**Explicit and implicit agency tasks.** (**A**) Explicit agency task: subjects were seated facing a computer screen and used their dominant hand to move the mouse, which drove a squared cursor along a horizontal line drawn at the bottom of the screen. Throughout all trial conditions, targets (X) and distractors (O) appeared at the top of the screen and moved down the screen at a consistent speed. Subjects were instructed to move the cursor to intercept the targets and to avoid the distractors when they arrived on the horizontal line. They were notified of a target (‘beep’) or a distractor (‘boop’) by specific audio feedback. Conditions were presented in 15-s blocks, repeated six times in a random order (6 × 4 blocks in total). The experiment included a training session of 1 or 2 controls blocks upon subject request. (**B, C**) Judgement phase of the explicit agency task. The participant clicked on the blue bar to report his or her judgement of control over the cursor’s movement (**B**). Likewise, the participant clicked on the red bar to report his or her judgement of goodness of performance (**C**). (**D**) The summary agency score for each experimental condition in the explicit agency task were compared between groups using ANOVA (19 patients with DYT-*SGCE* and 24 healthy volunteers). Groups differed in the Lag condition (*F*(1,42) = 26.04, *P* = 0.004). (**E**) Implicit agency task. Subjects were seated facing a computer screen, kept their hands on the keyboard and watched a 20-cm diameter clock on the screen. A dot appeared in random position on the circular border of the clock and travelled the circumference of the clock in a 6-s revolution time. In each trial, subjects were asked to press the space bar, resulting in stop of the dot which then disappeared. A marker appeared at a random position on the border of the clock, which could be moved around the clock with the left and right arrows. Participants returned the marker to its position when they had decided to press (‘intention’ condition) or pressed (‘action’ condition) the button. ‘M’ was a judgment (expressed in milliseconds) of the participants’ estimated marker position when they had pressed the spacebar compared to the real stop position of the dot. ‘W’ was a judgment (expressed in milliseconds) of the participants’ estimated marker position when they had decided to press the spacebar compared to the real stop position of the dot. ‘W-M’ is the difference (expressed in milliseconds) between the two. (**F**) Judgments for the ‘Action’ (judgment ‘M’) and ‘Intention’ (judgment ‘W’) conditions and overall judgment ‘W-M’ in the implicit agency task in milliseconds (ms) were compared between groups using ANOVA. DYT-*SGCE* , myoclonus dystonia; HV, healthy volunteers; DeltaJo, summary agency score; M, action block; W, intention block. *Significant results (*P* < 0.05). NS, non-significant results.

This task was adapted from that described in Metcalfe *et al*.^10,35,36^ (Fig. 1A–C). Subjects were seated facing a computer screen and used their dominant hand to move a mouse, which moved a squared cursor along a horizontal line at the bottom of the screen. In all trial conditions, targets (‘X’) and distractors (‘O’) appeared at the top of the screen and moved downwards at a constant speed. Subjects were instructed to move the cursor to intercept the targets as they arrived on the horizontal line, whilst avoiding the distractors. Four study conditions were used:^36^ (i) ‘Control’ condition, in which spatial and temporal concordance existed between subjects’ mouse movements and cursor position; (ii) ‘Turbulence’ condition, in which a random time interval was inserted between subjects moving the mouse and the resulting movement of the cursor on the screen; (iii) ‘Lag’ condition, in which a fixed 500 ms time interval was introduced between mouse movements and their effect on cursor position; (iv) ‘Magic’ condition, in which the size of the cursor field was increased by 10 pixels when approaching targets, facilitating target interception.

In the turbulence condition, the association between subject intention (mouse movement) and action outcome (cursor movement) was random. This artificially impaired subjects’ task performance, leading to a decreased SoA. This effect is relatively easy to detect, and the random delay prevents any adaptation of the system. In the Lag condition the time interval between action and outcome was constant, allowing subjects to adapt to the delay and improve their sense of control. The Lag condition, therefore, is expected to be less challenging and to have a lower impact on SoA than the Turbulence condition is the subjects can grasp the ‘rule’ related to the constant delay between the action and the actual outcome. In the Magic condition, the participant's performance is artificially inflated, creating a mismatch between the action and the outcome, and a match between the intention and the outcome. The Magic condition identifies individuals who place greater emphasis on assessing agency through the alignment of intention and outcome, as opposed to relying on the congruence between action and outcome (i.e. monitoring of the action). Subjects were not explicitly informed about the different task conditions. At the end of each trial, subjects indicated on a visual scale (i) their perceived control over the cursor through their mouse movements [raw judgment of agency (JoA)] and (ii) their perceived ability to reach targets and avoid distractors [judgment of performance (JoP)].

These two responses were then converted to numeric variables scored 0–100, and a summary agency score (DeltaJo) was calculated for each experimental condition using the following formula:^35^


$$\mathrm{DeltaJo}=(\mathrm{Jo}P_{c}-\mathrm{Jo}A_{c})-(\mathrm{Jo}P_{E}-\mathrm{Jo}A_{E}),$$

where ‘E’ and ‘C’ refer to the ‘Experimental’ condition and the ‘Control’ condition respectively. Computation of this composite score allows both (i) limiting of scaling effects by calculating experimental condition outcomes relative to the control condition and (ii) assessment of perceived control relative to perceived performance. This allows for the exploration of factors other than performance informing feelings of control, including action monitoring. Hence, these summary agency scores are more reliable than the JoA in identifying individuals who accurately perceive their loss of control over actions. These scores were considered as the primary explicit agency outcome in each experimental condition.^36^ Healthy subjects’ summary agency scores were typically 0 in the control condition, and negative in the three experimental conditions. This reflects participants’ recognition that their control was not solely based on their performance.^36^

#### Implicit agency task

As depicted in Fig. 1E, participants were seated in front of a computer, with their dominant hand on the computer keyboard. On the screen, they were presented with a white clock (diameter of 20 cm). On the peripheral part of this clock, a red dot appeared and started rotating on a circular trajectory parallel to the edge of the clock (the initial position of the dot appearance was randomized across trials). In each trial, participants had to press the keyboard space bar whenever they wish, causing the stop of the dot, which then disappeared. A marker then appeared at a random location on the clock face, which the subject instructed to move using the right and left arrow keys. There were two conditions divided in two blocks: (i) In the ‘action block’ the subject had to place the marker exactly where they thought the dot was when they had pressed the space bar. (ii) In the ‘intention block’, they had to place the marker where they thought the dot was when they had made the decision to press the space bar. In each trial, participants confirmed the marker's position by pressing the space bar, validating their responses. Each block consisted of 40 consecutive trials. To avoid attentional errors to instruction, the trials within each block belonged to the same condition and were not mixed. For each trial, the participant's estimated marker position was compared to the real stop position of the red dot. These judgment regarding the real position (expressed in milliseconds) were labelled as ‘M’ for the action block and ‘W’ for the intention block.

For each subject, values that were more than 3 SD from the mean were excluded in each block as outliers. The median value of ‘M’ and ‘W’ judgments for each subject was then calculated. We chose to compute the median value as it considered to be less sensitive to extreme values. Additionally, the overall judgment ‘W-M’ was calculated by subtracting ‘M’ from ‘W’. In summary, these three measures (‘W’, ‘M’ and ‘W-M’) constituted the outcomes analysed in the task.^37^ An ability to appropriately identify as the author of one's action is necessary to accurately assess the timing of the decision to initiate the action relative to the moment of the action initiation. A difference in the sense of agency can thus be explored by comparing these judgments (‘W’, ‘M’ and ‘W-M’) between the groups.^38^

### Imaging data acquisition, pre-processing and analysis

All scans were acquired using a 3T MRI scanner (Siemens Prisma, Germany) with a 64-channel head coil at the Paris Brain Institut neuroimaging platform. 3D T1-weighted MP2RAGE, a multi-shell diffusion tensor imaging and resting state functional images were acquired using a multi-echo echo-planar imaging (parameters provided in Supplementary materials).

#### Region of interest selection

**Figure 2:**
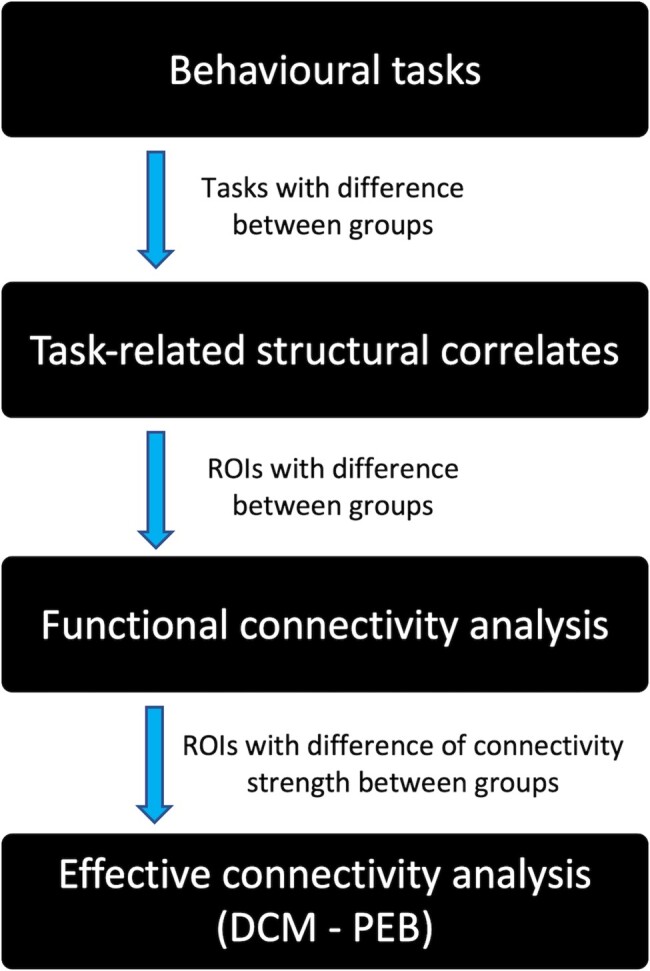
**Statistical analysis and data analysis plan.** We first analysed the behavioural data arising from the explicit and implicit agency tasks. Behavioural outcome measures showing significant differences between groups were considered for neuroimaging analysis. We assessed associations between behavioural markers of sense of agency and structural metrics in regions of interest (ROIs), which show alterations of grey matter and diffusion weighted imaging measures in patients. We then characterized the functional coupling (functional connectivity) between the ROIs which showed a degree of structural impairment related to altered sense of agency. Lastly using dynamic causal modelling (DCM) with Parametric Empirical Bayes (PEB), we evaluated the direction of the coupling (i.e. which area drives the activity of other ROIs).

We selected region of interests (ROIs) based on previous findings obtained using similar experimental paradigms.^9,10,12^ As shown in Fig. 2, we focused on the neural correlates of tasks which showed a significant difference in between-groups performance after analysis of behavioural results.

ROIs were arranged in three functional modules, as identified in the SoA literature:^9^ (i) a module associated with detection of action-outcome mismatch—pre-SMA, SMA proper and IPL; (ii) a module associated with action-outcome match detection—insula and precuneus and (iii) a sub-cortical module focused on action-outcome prediction—cerebellum and putamen; these regions have been previously associated with explicit SoA.^10^

The masks used for the analysis were generated using the Wake Forest University Pickatlas (http://fmri.wfubmc.edu/software/pickatlas). For the insula, the precuneus and the putamen we used masks derived from the automated anatomical labelling atlas.^39^ Pre-SMA and SMA proper ROIs were created based on the automated anatomical labelling SMA label and by dividing it into pre-SMA and SMA proper by drawing a vertical plane crossing the anterior commissure.^40^ For the cerebellum, we used the common mask for lobules IV, V and VI (automated anatomical labelling atlas) due to their connections with cortical motor areas.^41,42^ To explore the IPL, we built bilateral ROIs defined as spheres of 8 mm radius on the Montreal Neurological Institute coordinates (left IPL: −50, −50, 34; right IPL: 54, −50, 32), as previously performed when utilizing our explicit agency behavioural task.^10^

#### Voxel-based morphometry

Pre-processing was performed using Statistical Parametric Mapping software (SPM12; Wellcome Trust Center for Neuroimaging, UCL, London, UK) running MATLAB and Statistics Toolbox Release 2020a (The MathWorks, Inc., Natick, MA, USA).

We performed a voxel-based morphometry (VBM) analysis, as described in Statistical Parametric Mapping (SPM) Manual (https://www.fil.ion.ucl.ac.uk/∼john/misc/VBMclass15.pdf). Briefly, after segmentation of T1 images into grey, white matter and cortico-cerebral fluid images for each subject in native space, we used DARTEL to determine the non-linear deformations for wrapping inter-subject grey and white matter images. We focused on the analysis of grey matter images only. After spatial normalization of grey-matter images to a standard Montreal Neurological Institute space, smoothing (full width at half maximum [FWHM] = 10 mm) were applied. We extracted the total intracranial volume and the overall weighted image quality (IQR) using the Computational Anatomy Toolbox (CAT12, http://dbm.neuro.uni-jena.de/cat/) implemented in SPM12^43^ according to the toolbox manual (http://dbm.neuro.uni-jena.de/cat12/CAT12-Manual.pdf). Total intracranial volume and IQR were used as co-variables of non-interest for imaging analysis. We extracted the average signal intensity across the ROI for each subject using the Marsbar toolbox (http://marsbar.sourceforge.net).

#### Diffusion-weighted magnetic resonance imaging

Data pre-processing was performed using the Oxford University Centre for Functional MRI of the Brain (FMRIB) software library (FSL, https://fsl.fmrib.ox.ac.uk/fsl/fslwiki). After correction for motion and eddy currents, tensor reconstruction, non-linear registration, alignment to the Montreal Neurological Institute space, thresholding data at 0.2 and smoothing (FWHM = 5 mm), we performed the extraction of the average mean diffusivity (MD) across the ROI for each subject using Marsbar toolbox (http://marsbar.sourceforge.net). The absolute motion of each subject was extracted and used as a covariable of non-interest for imaging analysis.

#### Resting state functional MRI pre-processing

Echo planar imaging (EPIs) was processed with the TEDANA toolbox (v0.0.0a1, https://tedana.readthedocs.io/en/stable/). We used multi-echo imaging for resting state functional MRI pre-processing acquisition—an advanced method to efficiently perform movement corrections.^44^ The standard pre-processing steps were achieved using AFNI (v19.3.08 https://afni.nimh.nih.gov/pub/dist/doc/program_help/afni_proc.py.html), despiking, slice timing correction and motion correction (see Supplementary materials). The framewise displacement of each subject was extracted and used as a covariable of non-interest for imaging analysis. Then, TEDANA was used for realignment to the first volume driven by the first echo and co-registration to the anatomic volume. A single warp was applied to combine realignment and co-registration. A principal component analysis was applied to reduce the dimensionality of the dataset by removing thermal noise, and an independent component analysis decomposition to separate blood-oxygen-level dependent from non-blood-oxygen-level dependent components based on the echo time dependence of the blood-oxygen-level dependent component^45^ (non-blood-oxygen-level dependent components were visually inspected for each individual). After the independent component analysis, we obtained a dataset where both thermal noise and physiological noise such as movements, respiration artefacts and cardiac artefacts were removed. The data were normalized to the Montreal Neurological Institute template and smoothed (FWHM = 4 mm).

### Statistical analysis and data analysis plan

As presented in Fig. 2, we first analysed the behavioural data of explicit and implicit agency tasks. Behavioural outcome measures showing significant differences between groups were considered for neuroimaging analyses. We assessed the association between behavioural markers of SoA and those structural metrics in ROIs which showed alterations of grey matter, and diffusion-weighted imaging (DWI) measures in patients. We then characterized the functional coupling (functional connectivity) between the ROIs which showed a degree of structural impairment related to altered SoA. Lastly using dynamic causal modelling (DCM), we evaluated the direction of the coupling (i.e. determining which area drove the activity of other ROIs) to isolate areas that functionally led to altered connectivity.

### Statistical analysis of clinical and behavioural data

Statistical analysis was performed using the Statistical Package for Science (SPSS) version 21 (SPSS Inc. Chicago, IL, USA). We removed outliers from the behavioural tasks’ outcomes (Summary agency score -DeltaJo- for each condition for the explicit task, and judgment W-M for the implicit task) given the rarity of the pathology and the sample size, justifying the need to limit data heterogeneity. A permissive 3 SD cutoff was applied. Data from these two tasks were treated as independent measures. Comparison of behavioural outcome measures between the groups was performed using ANOVA models. For the tasks outcomes significantly differing between groups, Pearson correlations or partial correlations were performed to assess the relationships between the task outcome measures and clinical and imaging variables.

### Statistical analysis neuroimaging data

All imaging data analysis was performed with sex, age and total intracranial volume. Quality parameters (IQR, absolute motion and framewise displacement) were added as co-variables of non-interest according to the imaging dataset analysed. For all these analyses, *P* < 0.05 was assumed to be statistically significant.

For VBM and DWI, the signal extracted from the ROI was used to perform between-group comparisons using ANCOVA. For each ROI, we first performed a multivariate analysis including VBM and MD as variables in the same model. For ROIs which differed between groups in multivariate analysis, we performed a complementary univariate analysis to separately investigate specific VBM and MD differences between groups. The Bonferroni correction for multiple comparisons was applied within each functional module.

To explore functional connectivity, following the pre-processing steps described above, functional connectivity analysis was performed using the DPABI toolbox^46^ within MATLAB R2020a. We performed a ROI-to-ROI analysis for any ROI displaying structural abnormalities. ANCOVA was used to compare between groups the functional connectivity correlation coefficients for both (i) ROIs within the same functional module and (ii) ROIs belonging to other functional modules. The Bonferroni correction for multiple comparisons was performed where merited by the number of ROI in a target module. All corrected *P*-values are reported with the number of tests taken into account as a subscript, as follows: *p*_corr(*n* = ‘number of tests’)_.

For any ROI displaying significant differences during group comparisons using any imaging modality, partial correlation analyses with behavioural and clinical variables were then performed independently.

### Statistical analysis of effective connectivity

ROI which showed a between-groups difference in functional connectivity were included in DCM analysis utilizing a Parametric Empirical Bayes (PEB) methodology.^47,48^ We utilized a classical SPM approach to perform this analysis (see https://en.wikibooks.org/wiki/SPM/Parametric_Empirical_Bayes_(PEB)). A full model was constructed for each subject and used in PEB. Briefly, PEB estimates the connection parameters at the group level, then incorporates these group-level parameters into individual subjects’ DCMs as priors. These DCMs are then updated with these ‘empirical priors’ derived from the group to produce new posteriors. At the group level, we searched over nested PEB models to prune away any connectivity parameters from the PEB which were not contributive to the model. The connectivity parameters that we considered were intrinsic connectivity (auto-correlation within a ROI, with negative parameters being associated with excitatory influence, and positive parameters being associated with inhibitory influence) and extrinsic connectivity (between-ROIs causal correlation; with negative parameters being associated with inhibitory influence, and positive parameters being associated with excitatory influence). The PEB then provides updated results in terms of posterior probability (pp). We established high thresholds for estimated parameters (i.e. pp > 0.99) based on free energy. Those estimated parameters passing these thresholds were then considered to describe (i) commonalities across the whole group of participants and (ii) the differences between patients and HV in effective connectivity. Individual connection parameters updated from the PEB with group-level priors were used for independent partial correlation analysis with behavioural and clinical variables with sex, age, total intracranial volume and framewise displacement as co-variables of non-interest.

## Results

### Participants

Twenty-four patients (DYT-*SGCE*) and 25 healthy volunteers (HV) were included in the study. The results from one HV were outliers on the implicit agency task, and the subject was removed from the analysis. Additionally, five patients were excluded from the analysis due to insufficient imaging data quality or incomplete scanning procedures. As shown in Table 1, there were no statistically significant differences in demographic data between groups. DYT-*SCGE* patients showed more anxiety disorders compared to HV.

**Table 1:** Demographic and clinical data

	DYT-*SGCE* (*n* = 19)	HV (*n* = 24)	F (*P*)
Age^a^ (range)	30.37 ± 2.68 (19–60)	29.71 ± 2.15 (18–54)	0.04 (0.85)*
Years of education^a^	13.94 ± 0.35	13.17 ± 0.37	2.20 (0.16)*
Laterality (R:L)^b^	16:3	19:5	1.00**
Sex (F:M)^b^	11:8	14:10	1.00 **
BFM^a^	12.84 ± 2.63		
UMRS^a^	34.16 ± 4.06		
MDE/dysthymia^b^	3	0	0.08**
**Anxiety disorder^b^**	**15**	**2**	**<0**.001**
OCD^b^	5	1	0.07**
Addiction^b^	1	0	0.44**
**Medication^c^**	**9**	**0**	**<0**.001**

^a^Reported as mean ± Standard Error of the Mean.

^b^Reported in effective.

^c^Zonisamide (*n* = 4), Benzodiazepine (*n* = 4) Trihexyphenidyl (*n* = 1), Selective serotonin reuptake inhibitors (*n* = 2), Tetrabenazine (*n* = 1), Botulinic toxin injections (*n* = 2).

*ANOVA analysis.

**Fischer’s exact test.

Significant test results in bold.

DYT-*SGCE*, myoclonus dystonia; HV, healthy volunteers; R, righty; L, lefty; F, female; M, male; BFM, Burke-Marsden-Fahn scale; UMRS, Unified Myoclonus Rating scale; MDE, major depressive episode; OCD, obsessive-compulsive disorder.

### Explicit agency task

Before investigating the SoA, we first performed a between-groups comparison for all measures in the ‘Control’ condition of this task. The main interest was to investigate the relation between subjects’ judgment of performance and their actual performance to ensure that the groups do not differ in their perception of performance.^36^ The DYT-*SGCE* group intercepted a smaller number of target stimuli in the ‘Control’ condition compared to HV (mean ± Standard Error of the Mean (SEM), DYT-*SGCE*: 15.34 ± 0.55, HV: 17.88 ± 0.35, *F*(1,42) = 16.23, *P* = 0.0002) and had a lower raw judgment of performance score (JoP_c_) [mean ± SEM, DYT-*SGCE*: 69.60 ± 3.60, HV: 81.55 ± 2.15, *F*(1,42) = 8.90, *P* = 0.01]. There were no statistically significant differences in other measures (all *P* > 0.05) (Table 2). To assess subjects’ perception of their performance in this condition, we divided the number of targets hit by JoP_c_ and found no statistical difference between groups [*F*(1,42) = 0.36, *P* = 0.55].

**Table 2:** Explicit agency task results

	DYT-*SGCE* (*n* = 19)	HV (*n* = 24)	F (*P*)*
**Control condition**			
TrialMov^a^	2560,26 ± 180.82	2659.10 ± 106.93	0.24 (0.63)
**Targ_hit^a^**	**15.34** ± **0.55**	**17.88** ± **0.35**	**16.23** (**0.0002)**
JoA^a^	83.46 ± 3.05	87.45 ± 2.40	1.09 (0.30)
**JoP^a^**	**69.60** ± **3.60**	**81.55** ± **2.15**	**8.90** (**0.01)**
**Turbulence condition**			
TrialMov^a^	2797.04 ± 238.75	3160.70 ± 205.39	1.34 (0.25)
Targ_hit^a^	7.54 ± 0.30	8.08 ± 0.31	1.52 (0.23)
JoA^a^	27.98 ± 3.40	27.73 ± 3.40	0.003 (0.96)
JoP^a^	29.65 ± 2.87	30.41 ± 3.67	0.03 (0.88)
deltaJo^a^	−15.53 ± 3.75	−8.58 ± 2.14	3.73 (0.11)
**Lag condition**			
TrialMov^a^	4154.32 ± 461.94	5176.21 ± 526.59	2.01 (0.16)
Targ_hit^a^	5.57 ± 0.26	5.49 ± 0.32	0.03 (0.86)
JoA^a^	20.45 ± 3.39	23.22 ± 3.48	0.31 (0.58)
JoP^a^	22.64 ± 3.43	23.62 ± 3.34	0.04 (0.84)
**deltaJo^a^**	**−16.04 ± 4.01**	**−6.31 ± 2.05**	**26.04** (**0.004)**
**Magic condition**			
TrialMov^a^	2481.18 ± 124.96	2660.94 ± 87.30	1.47 (0.23)
Targ_hit^a^	19.67 ± 0.35	20.29 ± 0.23	2.43 (0.13)
JoA^a^	92.15 ± 2.04	91.33 ± 1.86	0.09 (0.77)
JoP^a^	86.67 ± 2.59	89.81 ± 1.82	1.04 (0.31)
deltaJo^a^	−5.18 ± 1.61	−3.40 ± 0.93	0.22 (0.66)

^a^Reported as mean ± Standard Error of the Mean.

*ANOVA analysis.

Significant test results in bold.

DYT-SGCE, myoclonus dystonia; HV, healthy volunteers; TrialMov, mean number of mouse movements per trial in pixels; Targ_hit, mean number of targets hit per trial; JoA, Mean judgment of agency per trial; JoP, mean judgment of performance per trial; deltaJo, mean summary of agency score per trial.

Next, we compared the SoA between groups. We performed an ANOVA to investigate between-groups differences in summary agency scores. We found an effect of group in the ‘Lag’ condition [*F*(1,42) = 26.04, *P* = 0.004], with a lower summary agency score (DeltaJo_Lag_) in DYT-*SGCE* (mean ± SEM: −16.04 ± 4.01) compared to HV (mean ± SEM: −6.31 ± 2.05) (Fig. 1D) identified through *post hoc* comparisons. There was no effect of group on other experimental conditions (all *P* > 0.1). There was no Group × JoP_c_ interaction in the ‘Lag’ condition [*F*(1,42) = 0.92, *P* = 0.38]. To ensure this significant finding could not be attributed to psychiatric pathology, we performed an additional ANCOVA with co-morbid anxiety disorders, obsessive-compulsive disorder and major depressive episodes as co-variables of non-interest. This did not alter the group difference in DeltaJo_Lag_ [*F*(1,42) = 8.60, *P* = 0.04]. Behavioural data are displayed in Table 2.

We explored the relationships between DeltaJo_Lag_ and clinical measures of movement disorder, including myoclonus severity (assessed using the UMRS scale) and dystonia (assessed using the BFM scale) using a partial correlation, in which JoP_c_ was added as a covariable of non-interest. No significant results were identified (*P* > 0.05).

### Implicit agency task

ANOVA was used to explore between-group differences in judgements, and no significant results were identified: M (mean ± SEM: DYT-*SGCE*: −34.65 ± 4.78; HV: −33.68 ± 3.42; *F*(1,42) = 0.03, *P* = 0.87), W (mean ± SEM: DYT-*SGCE*: −36 711 ± 92.03; HV: −297.57 ± 74.36; *F*(1,42) = 0.35, *P* = 0.56) and W-M (mean ± SEM: DYT-*SGCE*: −332.46 ± 90.37; HV: −263.89 ± 74.50; *F*(1,42) = 0.35, *P* = 0.56) (Fig. 1F).

### Neuroimaging results

#### Group differences in structural analysis: VBM and MD

A multivariate ANCOVA identified a significant between-group difference in morphometric measures for the motor cerebellum bilaterally (left cerebellum IV-V-VI: *F*(2,35) = 5.23, *p*_corr(*n* = 2)_ = 0.02; right cerebellum IV-V-VI: *F*(2,35) = 4.33, *p*_corr(*n* = 2)_ = 0.04; Table 3). No effect of group was identified for any other ROI (Table 3).

**Table 3:** Morphometric MRI data

		Multivariate				Univariate^b^
		F (*P*)*		DYT-*SGCE* (*n* = 19)	HV (*n* = 24)	F (*P*)**
**Mismatch detection functional module**						
Pre-SMA	Merged	1.53 (0.69)	VBM^a^	0.33 ± 0.01	0.31 ± 0.01	-
			MD^a^	0.0006 ± 0.00002	0.0007 ± 0.00001	-
SMA proper	Merged	2.49 (0.29)	VBM^a^	0.27 ± 0.01	0.25 ± 0.01	-
			MD^a^	0.0006 ± 0.00001	0.0006 ± 0.00001	-
IPL	Left	2.30 (0.31)	VBM^a^	0.30 ± 0.02	0.28 ± 0.01	-
			MD^a^	0.0003 ± 0.000003	0.0003 ± 0.000002	-
	Right	0.85 (1.00)	VBM^a^	0.36 ± 0.02	0.34 ± 0.01	-
			MD^a^	0.0004 ± 0.000004	0.0004 ± 0.000003	-
**Match detection functional module**						
Insula	Left	0.18 (1.00)	VBM^a^	0.38 ± 0.01	0.38 ± 0.01	-
			MD^a^	0.0006 ± 0.00001	0.0006 ± 0.000004	-
	Right	1.75 (0.38)	VBM^a^	0.42 ± 0.01	0.40 ± 0.01	-
			MD^a^	0.0007 ± 0. 00001	0.0007 ± 0.000005	-
Precuneus	Left	1.30 (0.57)	VBM^a^	0.31 ± 0.01	0.29 ± 0.01	-
			MD^a^	0.0006 ± 0.00001	0.0006 ± 0.00001	-
	Right	1.94 (0.32)	VBM^a^	0.31 ± 0.01	0.29 ± 0.01	-
			MD^a^	0.0006 ± 0.00001	0.0006 ± 0.00001	-
**Action-outcome prediction functional module**						
Putamen	Left	0.53 (1.00)	VBM^a^	0.30 ± 0.01	0.29 ± 0.01	-
			MD^a^	0.0005 ± 0.00001	0.0005 ± 0.000005	-
	Right	1.44 (0.50)	VBM^a^	0.30 ± 0.01	0.29 ± 0.01	-
			MD^a^	0.0005 ± 0.00001	0.0005 ± 0.000004	-
Cerebellum	Left	**5.23** (**0.02)**	VBM^a^	**0.49** ± **0.01**	**0.47** ± **0.01**	**10.76 (0.004)**
			MD^a^	**0.0006** ± **0.000005**	**0.0006** ± **0.000004**	**6.84 (0.03)**
	Right	**4.33** (**0.04)**	VBM^a^	**0.51** ± **0.01**	**0.49** ± **0.01**	**8.67 (0.01)**
			MD^a^	0.0006 ± 0.00001	0.0006 ± 0.000004	4.23 (0.09)

^a^Reported as mean ± Standard Error of the Mean.

^b^Test provided only if difference in multivariate analysis.

*ANCOVA analysis (*P* corrected with Bonferroni correction for multiple comparisons) with age, sex, total intracranial volume, quality parameters (weighted overall image quality and absolute motion) as co-variables of non-interest.

**ANCOVA analysis (*P* corrected with Bonferroni correction for multiple comparisons) with age, sex, total intracranial volume and quality parameters (weighted overall image quality for voxel-based morphometry or absolute motion for MD) as co-variables of non-interest.

Significant test results in bold.

DYT-SGCE, myoclonus dystonia; HV, healthy volunteers; pre-SMA, pre-supplementary motor area; SMA, supplementary motor area; IPL, inferior parietal lobule; ROI, region of interest; VBM, voxel-based morphometry; MD, mean diffusivity.

**Figure 3:**
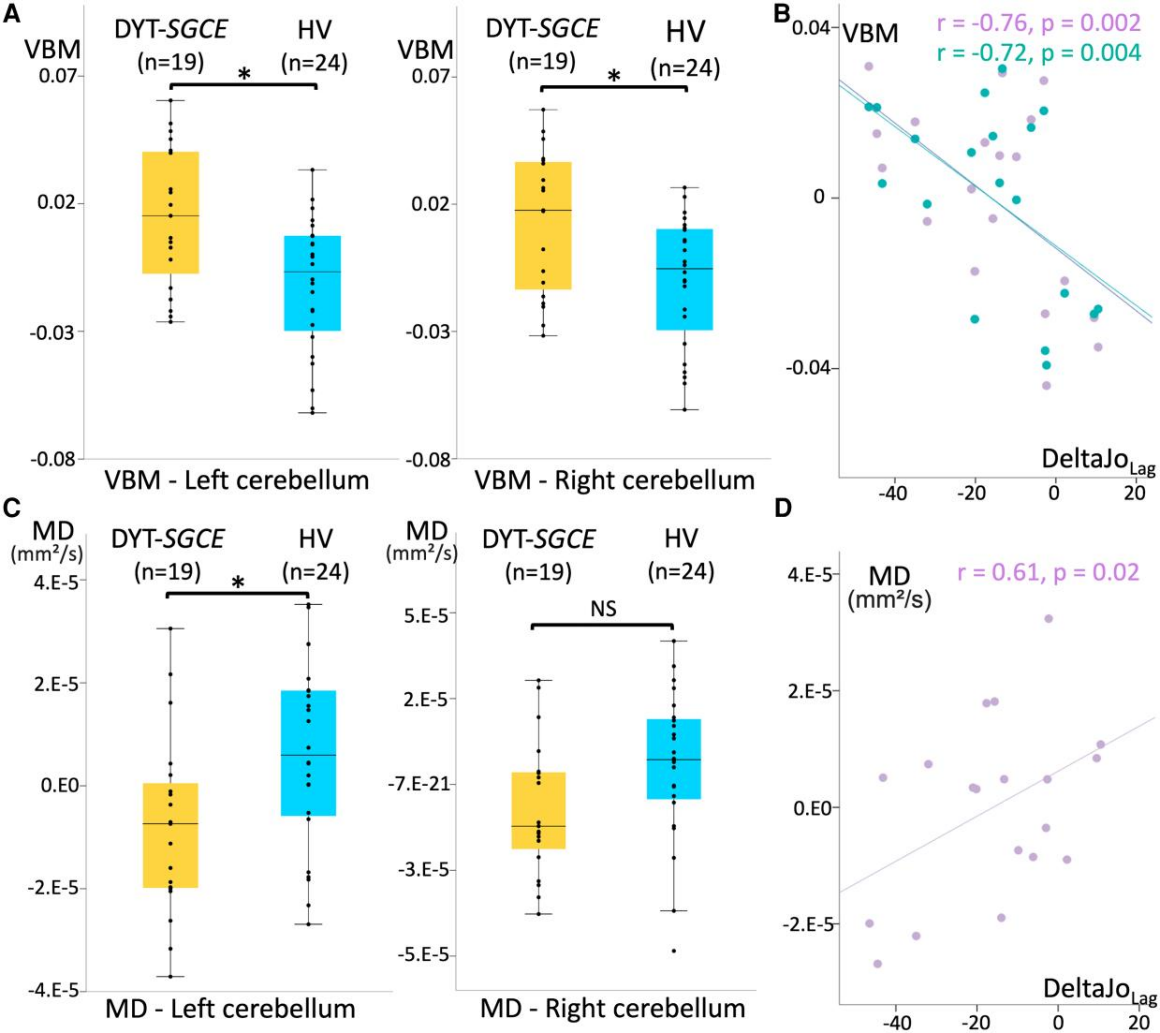
**Structural neuroimaging results.** (**A**) Results of region of interest voxel-based morphometry analysis of cerebellum IV, V and VI grey matter. Groups were compared using ANCOVA adjusted for age, sex, total intracranial volume and weighted image quality (19 patients with DYT-*SGCE* and 24 healthy volunteers). Groups differed for the left cerebellum IV-V-VI (*F*(1,37) = 10.76, *p*_corr_ = 0.004) and the right cerebellum IV-V-VI: *F*(1,37) = 8.67, *p*_corr_ = 0.01). (**B**) Partial correlation accounting for the effect of age, sex, total intracranial volume, weighted image quality and the judgment of performance in the ‘control’ condition during the explicit agency task in DYT-*SGCE* group of individual left (in purple, *r* = −0.76, *P* = 0.002) and right (in green, *r* = −0.72, *P* = 0.004) cerebellum IV, V and VI voxel-based morphometry signals on *y*-axis (corrected for covariable of non-interest) and the summary agency scores in the ‘Lag’ condition on *x*-axis. (**C**) Results of region of interest MD analysis of cerebellum IV, V and VI grey matter. Groups were compared using ANCOVA adjusted for age, sex, total intracranial volume and absolute motion (19 patients with DYT-*SGCE* and 24 healthy volunteers). Groups differed for the left motor cerebellum (*F*(1,37) = 6.84, *p*_corr_ = 0.03). (**D**) Partial correlation accounting for the effect of age, sex, total intracranial volume, absolute motion, and the judgment of performance in the control condition during the explicit agency task in DYT-*SGCE* group of individual left (in purple, *r* = −0.61, *P* = 0.02) cerebellum IV, V, and VI MD on *y*-axis (corrected for covariable of non-interest) and the summary agency scores in the ‘Lag’ condition on *x*-axis. DYT-*SGCE*, myoclonus dystonia; HV, healthy volunteers; VBM, voxel-based morphometry; MD, diffusion tensor imaging mean diffusivity. DeltaJo_Lag_ = summary agency score in the ‘Lag’ condition. *Significant results (*P* < 0.05). NS, non-significant.

Univariate analysis (Table 3; Fig. 3) revealed a higher T1 grey-matter signal bilaterally in the cerebellum (left cerebellum IV-V-VI: *F*(1,37) = 10.76, *p*_corr(*n* = 2)_ = 0.004; right cerebellum IV-V-VI: *F*(1,37) = 8.67, *p*_corr(*n* = 2)_ = 0.01), as well as lower MD in the left motor cerebellum (left cerebellum IV-V-VI: *F*(1,37) = 6.84, *p*_corr(*n* = 2)_ = 0.03) in DYT-*SCGE* compared with HV.

#### Structural correlates of task performance and symptoms severity

Correlation between summary agency score in the Lag condition of the explicit agency task (DeltaJo_Lag_) and those structural measures differing between groups are presented in Fig. 3. Importantly, we added JoP_c_ as a covariable of non-interest to this correlation as it differed significantly across groups. In the DYT-*SGCE* group, DeltaJo_Lag_ was both negatively correlated with T1 grey-matter signal in the bilateral cerebellum (left cerebellum IV-V-VI: *r* = −0.76, *P* = 0.002; right cerebellum IV-V-VI: *r* = −0.72, *P* = 0.004), and positively correlated with MD signal amplitude in the left cerebellum (*r* = 0.61, *P* = 0.02). Voxel-based morphometry and MD signals in the motor cerebellum did not correlate with clinical scores.

#### Resting-state functional connectivity analysis

**Figure 4:**
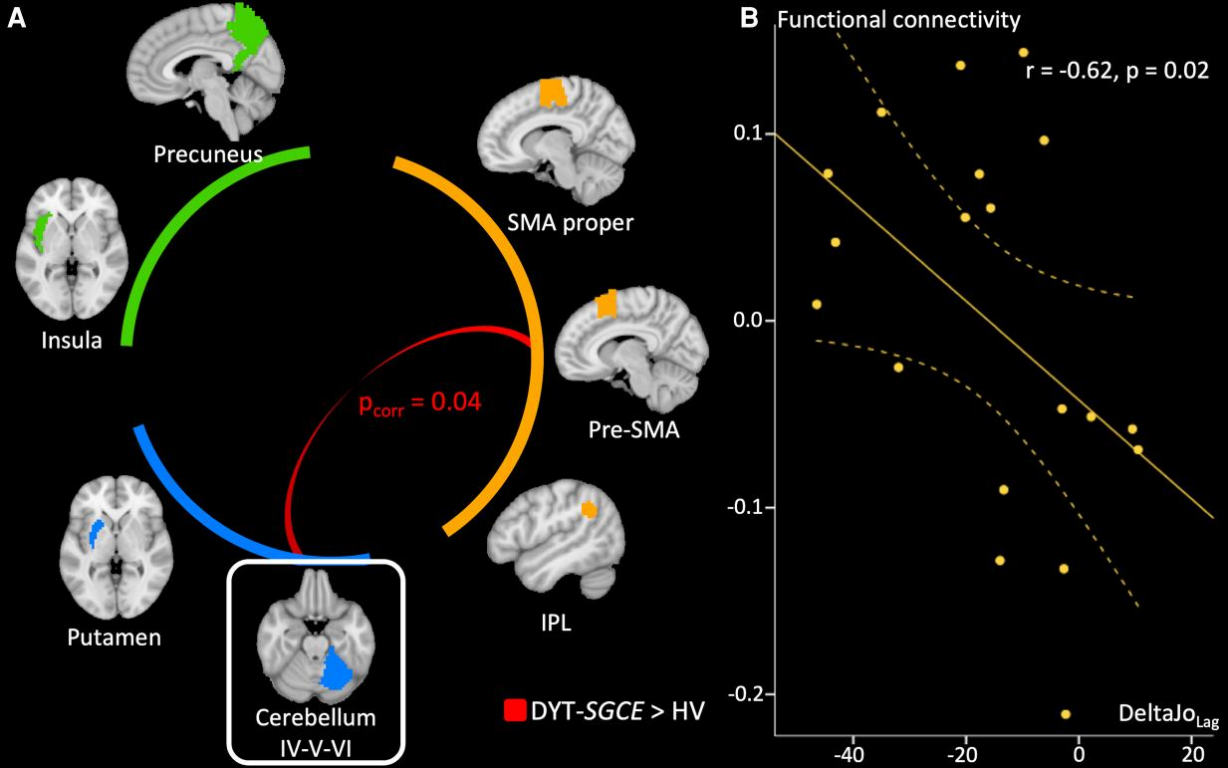
**Functional connectivity results.** (**A**) Results of the functional connectivity analysis group comparison based on correlation between the signal extracted in regions of interest (ROIs). Groups were compared using ANCOVA adjusted for age, sex, total intracranial volume and framewise displacement. ROIs were categorized into three functional modules: (i) a module associated with action-outcome mismatch detection comprising the pre-supplementary motor area, the supplementary motor area and the inferior parietal lobule, (ii) a module associated with action-outcome match detection comprising the precuneus and the insula, (iii) a module associated with action-outcome prediction comprising the putamen and the cerebellum IV,V,VI. Only connections from the right motor cerebellum (framed) were considered since it was the only ROIs which presented structural differences between groups. Stronger connections in patients than in healthy volunteers are displayed. *P*-value are corrected for multiple comparison with Bonferroni method within each functional module. The connectivity between pre-supplementary motor area and right motor cerebellum differed between groups (*F*(1,37) = 6.90, *p*_corr_ = 0.04). (**B**) Partial correlation accounting for the effect of age, sex, total intracranial volume, framewise displacement and the judgment of performance in the ‘control’ condition during the explicit agency task in DYT-*SGCE* group with 95% Confidence Interval (CI) of functional connectivity strength between motor cerebellum and pre-supplementary motor area on *y*-axis (corrected for covariable of non-interest) and the summary agency score in the ‘Lag’ condition in the explicit agency task on *x*-axis. DYT-*SGCE*, myoclonus dystonia; HV, healthy volunteer; IPL, inferior parietal lobule; pre-SMA, pre-supplementary motor area; DeltaJo_Lag_, summary agency score in the ‘Lag’ condition.

We explored functional connectivity through pair-wise correlation of ROIs across time using ANCOVA. As presented in Fig. 4A, this revealed significant functional connectivity between the right motor cerebellum and the pre-SMA [*F*(1,37) = 6.90, *p*_corr(*n* = 3)_ = 0.04]. Connectivity analysis found no other significant associations between either the left or right motor cerebellum and other ROI.

#### Resting-state effective connectivity analysis

**Figure 5:**
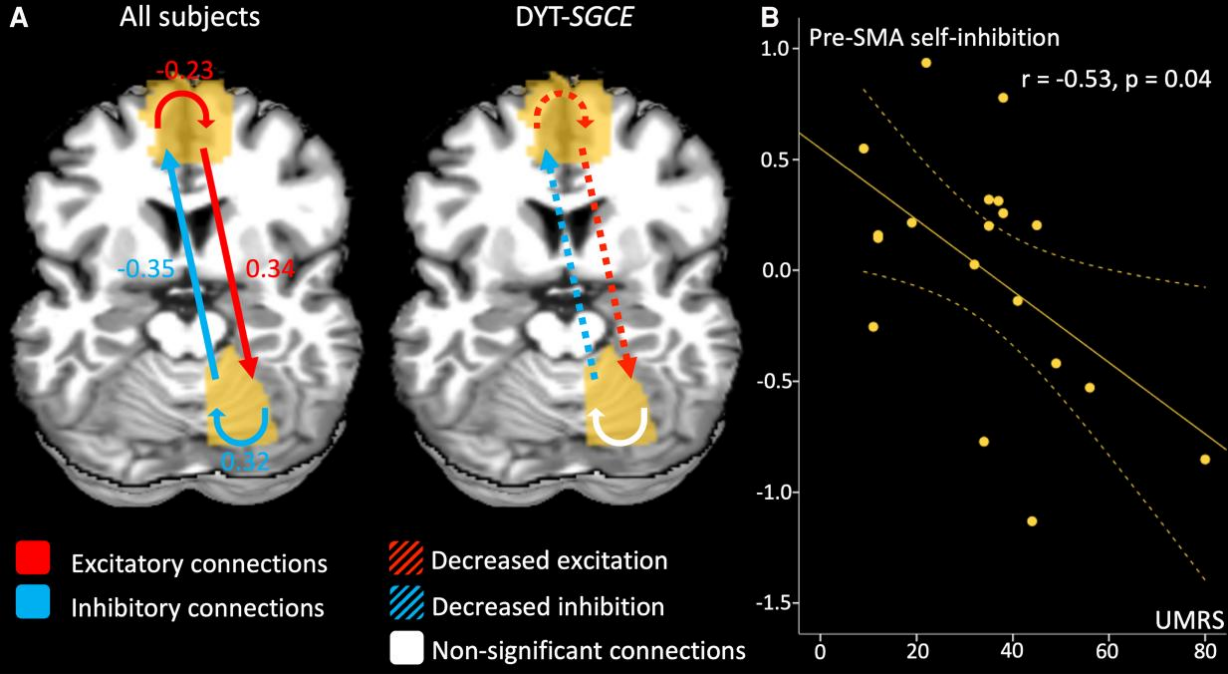
**Dynamic causal model effective connectivity results.** (**A**) The contribution of different parameters to model evidence across all subjects (left) and between groups (right). Parametric Empirical Bayes (PEB) employs Bayesian statistics. Consequently, the outcomes do not rely on *P*-values but are presented in terms of posterior probabilities (pp). The connections with a posterior probability (pp) > 0.99 are displayed in colour whereas the non-relevant connections are displayed in white. For the contrast of the whole group of participants, the effect size of the connectivity parameters are displayed. For between-region parameters (in Hz), positive numbers indicate excitation and negative numbers indicate inhibition. For self-connection parameters (no units), positive numbers indicate self-inhibition and negative numbers indicate disinhibition. These effects between regions and within regions are summarized by red arrows for excitatory connection and blue arrow for inhibitory connections. For the between groups contrast, we displayed the connections that differentiate groups in colour. Since the contrast patients > healthy volunteers indicate different outcomes depending on if parameters are positive or negative, we chose to summarize the results as follows: the red dotted arrows represent decreased excitatory connections, and blue dotted arrows represent decreased inhibitory connections. (**B**) Partial correlation accounting for the effect of age, sex, total intracranial volume, framewise displacement in DYT-*SGCE* group with 95% CI of self-inhibition within pre-supplementary motor area on *y*-axis (corrected for covariable of non-interest) and Unified Myoclonus Rating Scale on *x*-axis (*r* = −0.53, *P* = 0.04). DYT-*SGCE*, myoclonus dystonia; pre-SMA, pre-supplementary motor area; UMRS, Unified Myoclonus Rating Scale.

We further explored the abnormal functional connectivity identified between the right motor cerebellum and the pre-SMA using DCM (Fig. 5A and Supplementary Fig. 1). When exploring identified common findings across both participant groups, we found an inhibitory connection from the cerebellum to the pre-SMA, and an excitatory connection from the pre-SMA to the cerebellum. Intrinsic connectivity within cerebellum and pre-SMA acted as self-inhibition and self-disinhibition, respectively. Reciprocal connections (in both directions) between the pre-SMA and the cerebellum differed between the patient and HV groups, as did the self-disinhibition (intrinsic connectivity) within the pre-SMA. In the DYT-*SGCE* group, there was both a decreased inhibitory effect of the cerebellum on the pre-SMA, and a decreased excitatory effect of the pre-SMA on the cerebellum. Finally, those in the DYT-*SGCE* group showed reduced self-excitation within pre-SMA.

#### Functional correlates of task performance and symptoms severity

We performed correlation analyses between summary agency score in the explicit agency task in the Lag condition (the DeltaJo_Lag_ variable) and degree of functional connectivity. In the DYT-*SGCE* group, DeltaJo_Lag_ was negatively correlated with the degree of functional connectivity between the right motor cerebellum and the pre-SMA (*r* = −0.62, *P* = 0.02) (Fig. 4B). Degree of functional connectivity did not correlate with clinical scores. DCM parameters did not correlate with task performance, but pre-SMA self-inhibition strength negatively correlated with UMRS (*r* = −0.53, *P* = 0.04), as depicted in Fig. 5B.

## Discussion

DYT-*SGCE* patients exhibited a specific impairment in an explicit agency task when a constant time interval was imposed between their actions and outcomes. Neuroimaging analysis revealed both grey-matter abnormalities in the motor cerebellum and increased connectivity between the motor cerebellum and pre-SMA in patients compared to controls. The rest of the network involved in the sense of agency remained intact. The presence of these structural and functional abnormalities was also associated with a diminished sense of control in the explicit agency task. Additionally, DYT-SGCE patients displayed the following: (i) a decreased inhibitory effect of the cerebellum on the pre-SMA; (ii) a reduced excitatory effect of the pre-SMA on the cerebellum; and (iii) a decrease in self-disinhibition within the pre-SMA, which correlated with the severity of myoclonus. Consequently, our study expands the understanding of neurological dysfunction in DYT-*SGCE* by identifying the disruption of a high-level cognitive process.

This study had several limitations. Firstly, the correlational design employed prevents us from establishing a causal relationship between cerebellar dysfunction and altered sense of agency or symptom severity. Moreover, our neuroimaging results could be influenced by factors other than the SoA. For instance, the high prevalence of psychiatric comorbidities among our patients may have affected the behavioural or neuroimaging outcomes. Nevertheless, given that up to two-thirds of patients with DYT-*SGCE* experience co-morbid psychiatric conditions, our findings remain relevant and representative of this patient group.^21^ Furthermore, we attempted to account for potential biasing effects by limiting the brain regions we investigated to focus on those linked to the sense of agency and by controlling for spurious and nuisance covariates in the statistical models.

Secondly, no association was found between behavioural or neuroimaging measures and dystonia severity in DYT-*SGCE*. Previous evidence suggests that cerebellar dysfunction contributes to both myoclonus and dystonia in this disorder, as observed in mouse models where acute knockout of *SGCE* in the cerebellum resulted in both dystonia and myoclonic jerks.^49^ However, dystonia in DYT-*SGCE* is typically mild and focal, leading to a limited range of dystonia severity scores when using existing scales.^50^ This may have hindered the identification of any correlation between dystonia severity and neuroimaging or clinical parameters.

###  

#### Cerebellar structural and functional abnormalities and SoA in DYT-*SGCE*

In the control condition of the explicit agency task, the movements of the cursor precisely matched the movements of the mouse, enabling healthy participants to accurately intercept targets and avoid distractions. In this condition, patients were less performant than HV, likely due to their motor disability, and exhibited a lower JoPc consistent with their actual motor performance. In other words, they perceived their actual performance with the same accuracy than HV. This indicated the absence of a defect in the evaluation of the performance *per se*.^36^ However, introducing a delay between the cursor and the mouse movements disrupted the alignment between the participants’ actions and their outcomes, resulting in a perceived loss of control. In the Turbulence condition, this delay was randomly applied, while in the Lag condition, the delay was constant. Consequently, in the Lag condition, participants had the opportunity to capitalize on this implicit ‘rule’ to improve their sense of control over the task.^36^ Patients with DYT-*SGCE* felt less control in the Lag condition, while both groups experienced a similar loss of control during the Turbulence condition. This difference likely arose from DYT-*SGCE* patients’ inability to recognize the constant delay in the Lag condition and consequently, they were unable to take advantage of it to enhance their sense of control.

The motor cerebellum (lobules IV, V, and VI) is part of the cerebello-thalamo-pre-SMA-red nucleus loop.^51^ This loop is known to play a role in the early phase of movement learning, contributing to the development of optimal motor commands.^52^ The forward internal model proposes that the cerebellum acts as a predictive controller, generating internal models that anticipate the body's movements and their expected consequences. When the actual outcome of a movement deviates from the cerebellum's prediction, it sends a feedback signal to the motor system to adjust the motor command.^41,42,53-55^ The cerebellum is particularly involved in adjusting the temporal component of high-level predictions.^56^ In the Lag condition of the task, it is plausible that the cerebellum had an informative influence on the pre-SMA, enabling adjustments to account for the temporal delay between action and its outcome. This adjustment likely contributed to an increase SoA in HV. On the other hand, during the Turbulence condition, where the delay was random, the cerebellum was unable to effectively adjust its prediction of the action outcome. This mismatch between predicted and actual outcomes resulted in a change in the perception of agency, leading to a diminished feeling of control. Moreover, the SoA was selectively impaired in the Lag condition when this adaptive process of action-outcome prediction is required for motor adaptation. Patients with DYT-*SGCE* showed no alteration in their SoA in the Magic condition, indicating that they do not solely rely on the result of the action to construct their SoA but rather use the monitoring of their actions. However, no dynamic adaptation of the system is engaged in this condition. Similarly, in the implicit Libet task, there is no discrepancy between action and its outcome, or adaptive process involved. Awareness of action in cerebellar patients may persist, but it could be altered when adjustments are needed.^57^ This specific alteration of SoA in the Lag condition of the explicit task aligns with the theory that the cerebellum and its forward internal model play a major role in the abnormal SoA in DYT-*SGCE*.

DYT-*SGCE* patients were found to have grey-matter abnormalities in the motor cerebellum, which were associated with reduced explicit SoA in Lag condition. On a microarchitectural level, increased VBM signal might account for altered cerebellar maturation leading to an increased volume of neuropil. This finding is in line with recent observations describing an increased number of branches and longer branch lengths in dendrites in cortical cells in subjects with *SGCE*-mutation.^30^ Indeed, greater microstructural complexity in the entanglement of cytoplasmic protrusions from neurons (e.g. axons and dendrites) and glial cells in the extracellular space may constrain water diffusivity,^58-60^ consistent with the decreased MD we observed in DYT-*SGCE* patients. Changes to neurite morphology has been associated with diminished synaptic efficiency, might result from alteration of the neuronal homeostasis and should be involved in multiple neurodevelopmental disorders.^61-64^ An alternative proposal may be that those cortical neurons connected to the cerebellum are selectively affected by neuronal dysfunction (diaschisis). This is assumed in other developmental conditions, such as autism.^65^ This has been suggested to result in decreased MD.^66,67^ Future work should explore this hypothesis through techniques allowing better identification of the cerebellar neuropil microarchitecture, such as NODDI imaging.^68^

This work also investigated at rest the functional connections between the brain regions related to the SoA. In DYT-*SGCE*, we have identified abnormal excitatory and inhibitory connections between the motor cerebellum and pre-SMA, as well as showed a reduction in self-disinhibition within the pre-SMA itself. Previous work has shown that, via the cerebello-thalamo-cortical pathway, the cerebellum influences excitation and inhibition in the motor cortex.^69^ In other forms of dystonia, such as DYT-*TOR1A*, structural abnormalities in the cerebellar outflow pathway have been associated with increased activity within the pre-SMA during motor sequence learning, indicating the reduced inhibitory influence of the cerebello-thalamo-cortical pathway.^14^

Furthermore, our study revealed a decreased in self-excitation within the pre-SMA during rest. Previous research has found a lower membrane excitability of the corticocortical axons in individuals with *SGCE* mutation.^24^ We also observed that patients with milder myoclonus symptoms exhibited a greater decrease in self-excitation within the pre-SMA, implying a potential compensatory mechanism. However, the meaning of this finding remains to consider with caution.

We suggest that an imbalance between cerebellar-related excitatory and inhibitory signals might be a crucial factor contributing to the abnormal sense of agency and motor symptoms observed in DYT-*SGCE*, acknowledging that our study design is limited to infer any causal links.

## Supplementary Material

fcae105_Supplementary_Data

## Data Availability

The data supporting the findings of this study are available from the corresponding author, upon reasonable request.
