# Supplementary material for: Association of abnormal explicit sense of agency with cerebellar impairment in myoclonus-dystonia
Source: Brain Commun. 2024 Mar 27;6(2):fcae105. doi: 10.1093/braincomms/fcae105 (PMC11004927; doi:10.1093/braincomms/fcae105)
Supplement: fcae105_Supplementary_Data [file fcae105_supplementary_data.pdf]

# Supplementary data

## Supplementary materials:

### Power parameters

The number of participants was fixed according to a previous study utilizing our explicit agency behavioral task in cervical dystonia.[1] Using an ANOVA with an effect-size of f = 0.43 and with alpha = 0.05, 46 subjects were required to obtain a power of 80% (G-Power, v3.1).

### Behavioural tasks

The order of inclusions over time was balanced between patients and healthy volunteers and the order of the behavioral tasks was random among participants.

-The explicit agency task was developed on an MacOS environment. The task comported 15-second blocks, repeated six times in a random order (6x4 blocks in total). After each block, subjects rated their feeling of control and their feeling of performance. The total duration of the task was about 8 minutes depending on the subject.

-The implicit agency task was programmed using EventIDE software (OkazoLAB, Netherland, 2017). Each block included 40 consecutive trials. In each trial, the experimental conditions were presented in a constant order ("action" then "intention") to facilitate understanding of the task. The duration of the task was about 10 minutes depending on the subject.

### Imaging data acquisition

Whole brain scans were acquired on the 3T MRI scanner (Siemens Prisma, Germany) with a 64-channel head coil at the ICM neuroimaging platform.

3D T1-weighted MP2RAGE sequence was acquired in sagittal orientation, at 1 mm isotropic voxels resolution, with an in-plane matrix of 232 x 256 and 176 slices, TR = 5000 ms, TE = 2.96 ms, TI = 700 / 2500 ms, flip angle = 4 / 5°, grappa acceleration factor 3. Dummy scan were automatically discarded by the sequence, fat signal was suppressed. The head movements were video-tracked during the image acquisition, using the eye-tracking video system.

A multi-shell diffusion tensor imaging (DTI) acquisition was also performed (TR = 3.5s, TE = 75ms, Multi-band of 3, isotropic voxel size 1.75mm, 60 direction with b = 2000 s/mm2, 32 direction with b = 1000s/mm2 and 8 direction with b = 300s/mm2, one b = 0 was each 10 directions).

Resting state functional images were acquired using a multi-echo echo-planar imaging (EPI) sequence (350 time points), with a multi-slice, multi-echo acquisition scheme (TR = 1.9s, TE = 17/36/56ms, iPAT acceleration factor 2, Multi-band 2, isotropic voxel size 3mm, phase encoding direction P>A). The shim box was equal to the acquired volume. An interleaved ascending (MultiSlice) slide order was used during scans acquisition. Scans were acquired with the eyes opened with fixation on cross. The subjects were monitored with eye tracker system to assure that they were not somnolent during data acquisitions.

## DTI preprocessing detail

The 4D DWI raw data were first denoised and degibs[2] with mrtrix software. The B0 files with the Two-phase directions (AP-PA) were then used to correct for EPI susceptibility deformation with topup tool from fsl.[3] Topup compute the nonlinear spatial deformation constrained only along the phase encode direction. This deformation was then applied by eddy tool from FSL[3,4] which correction at the same time for subject motion, and apply the motion to the Diffusion gradient directions. Volumetric change are applied with the use of the Jacobian determinant of the deformation ( --resamp=jac options of eddy)

Finally, with use the automatic outlier detection implement in eddy with the -repol option.[5] The correction B=0 volume was then use to rigidly coregister the volume to the anatomical scan. After those preprocessings, we fit the Diffusion tensor model with dti_fit from FSL. The mean diffusivity is then normalized to the MNI space using the normalization computed with the T1 anatomical volume (spm_normalize function).

## Resting state functional MRI preprocessing detail

Concerning the scanner-side preprocessing: the reconstructed matrix was equal to the acquired matrix, there was no prospective motion correction, we used a signal inhomogeneity correction (option: "Prescan Normalize"), there was no distortion-correction. The preprocessing was performed using AFNI.[6,7] For slice-timing correction, the reference slice was the first slice (t=0), we used a heptic (7th order) Lagrange polynomial interpolation. The motion correction was performed using rigid body transformations, without susceptibility correction, the reference scan was fixed with the option "MIN_OUTLIER in afni_proc.py". The functional-structural co-registration was performed using rigid body transformations, normalized mutual information and the 4th degree B-Spline interpolation method. There was no intensity correction for BOLD. The despiking step was performed using the AFNI 3dDespike function.

# Supplementary results

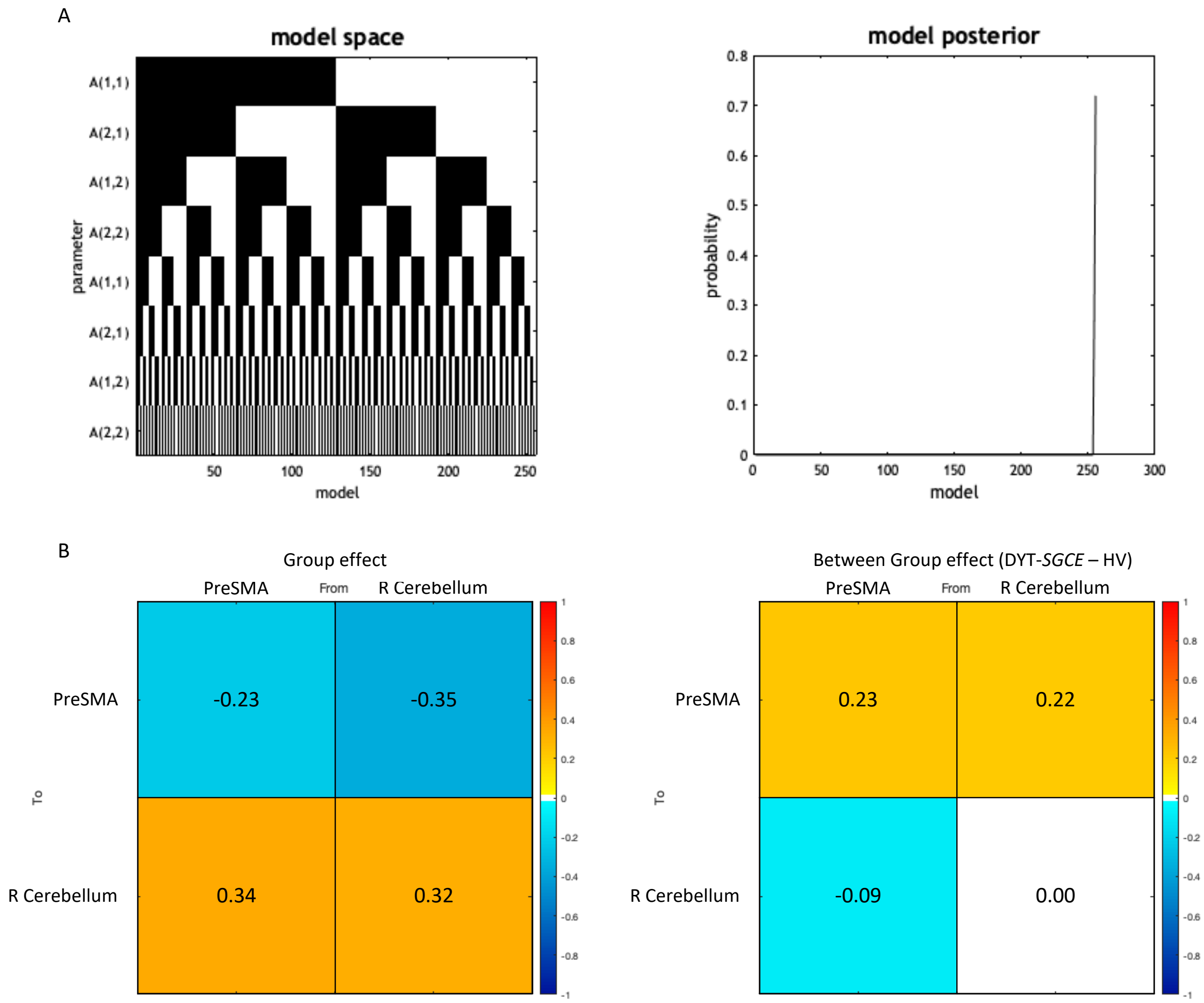


**Supplementary Fig.1 Detailed Bayesian dynamic model analysis**

A. The left chart shows all combinations of switching off parameters from the Parametric Empirical Bayes (PEB) model performed by the search algorithm. The rows are connections which varied between the models and the columns are the models. White means a connection switched on, and black means a connection switched off. The right panel shows the posterior probability of the final iteration of this search. The posterior probability of the best model found by this search was P = 0.72.

B. Matrix A of each parameter in the model after Bayesian model averaging. The left and the right charts represent respectively the effect of the entire group of participants and the between-group effect. Values and color represent the effect-size of each parameter. PEB employs

Bayesian statistics. Consequently, the outcomes do not rely on p-values but are presented in terms of posterior probabilities (PP). The connections with a posterior probability (PP) > 0.99 are displayed in color whereas the non-relevant connections are displayed in white.

## References


1. Delorme C, Roze E, Grabli D, et al. Explicit Agency in Patients with Cervical Dystonia: Altered Recognition of Temporal Discrepancies between Motor Actions and Their Feedback. *PloS One*. 2016;11(8):e0162191.

2. Tournier JD, Smith R, Raffelt D, et al. MRtrix3: A fast, flexible and open software framework for medical image processing and visualisation. *NeuroImage*. 2019;202:116137.

3. Andersson JLR, Skare S, Ashburner J. How to correct susceptibility distortions in spin-echo echo-planar images: application to diffusion tensor imaging. *NeuroImage*. 2003;20(2):870-888.

4. Andersson JLR, Sotiropoulos SN. An integrated approach to correction for off-resonance effects and subject movement in diffusion MR imaging. *NeuroImage*. 2016;125:1063-1078.

5. Andersson JLR, Graham MS, Zsoldos E, Sotiropoulos SN. Incorporating outlier detection and replacement into a non-parametric framework for movement and distortion correction of diffusion MR images. *NeuroImage*. 2016;141:556-572.

6. Cox RW. AFNI: Software for Analysis and Visualization of Functional Magnetic Resonance Neuroimages. *Comput Biomed Res*. 1996;29(3):162-173.

7. Cox RW, Hyde JS. Software tools for analysis and visualization of fMRI data. *NMR Biomed*. 1997;10(4-5):171-178.
